# Supplementary material for: Seminoma and Embryonal Carcinoma Footprints Identified by Analysis of Integrated Genome-Wide Epigenetic and Expression Profiles of Germ Cell Cancer Cell Lines
Source: PLoS One. 2014 Jun 2;9(6):e98330. doi: 10.1371/journal.pone.0098330 (PMC4041891; doi:10.1371/journal.pone.0098330)
Supplement: File S1 — ZIP file containing DMRforPairs output for significant regions. Please start from the html files. (ZIP) [file pone.0098330.s008.zip › figures/22931.pdf]

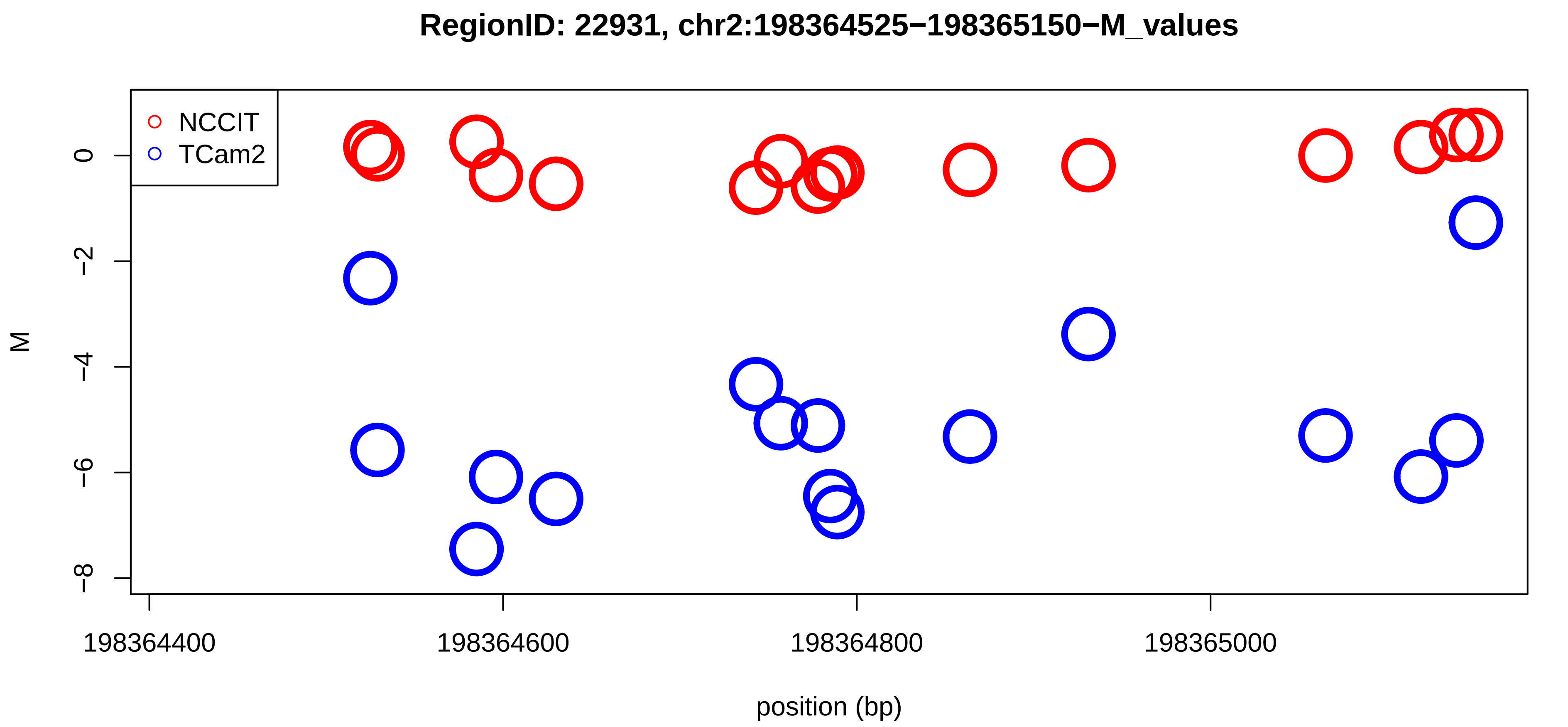
RegionID: 22931, chr2:198364525−198365150−M_values
NCCIT
TCam2
M
0
−2
−4
−6
−8
198364400
198364600
198364800
198365000
position (bp)

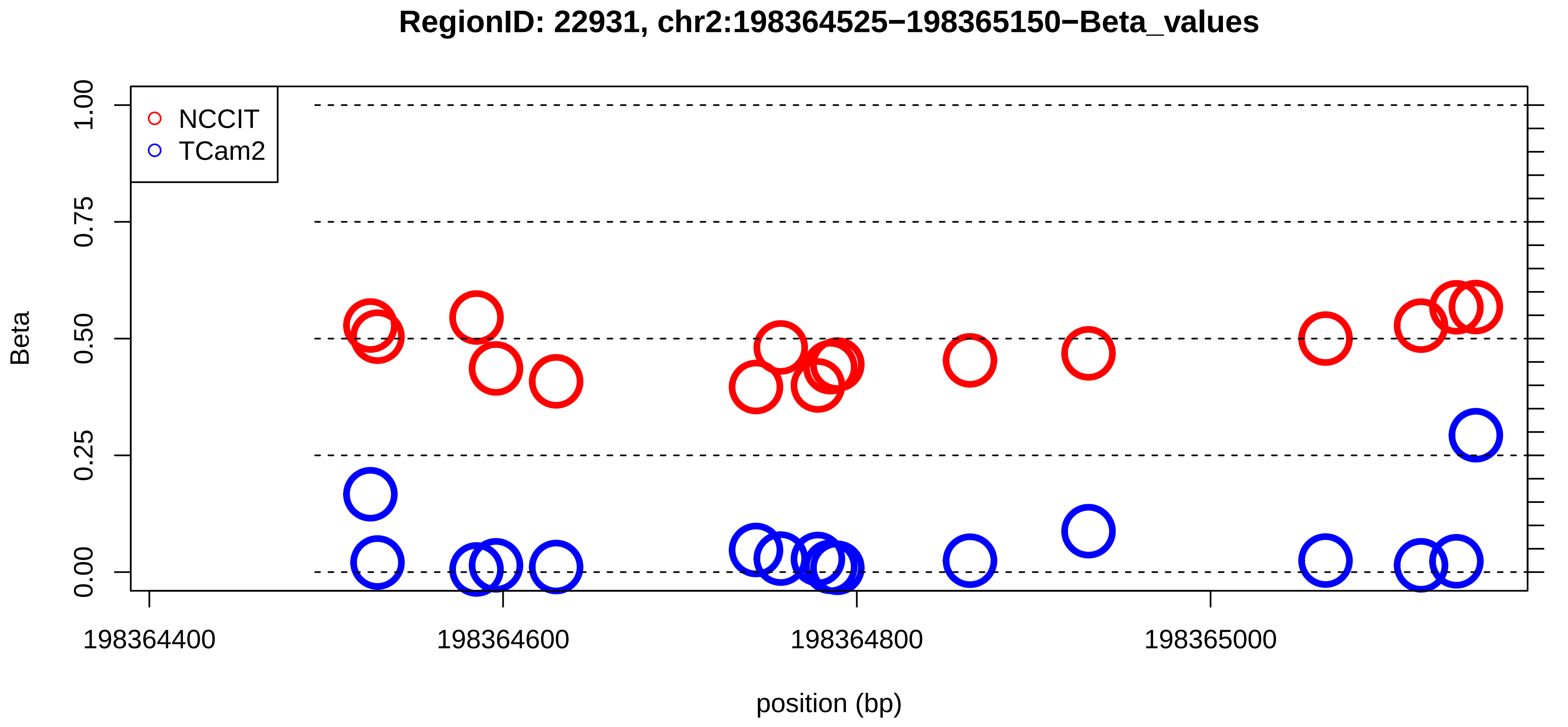
RegionID: 22931, chr2:198364525−198365150−Beta_values
NCCIT
TCam2
Beta
0.00
0.25
0.50
0.75
1.00
198364400
198364600
198364800
198365000
position (bp)

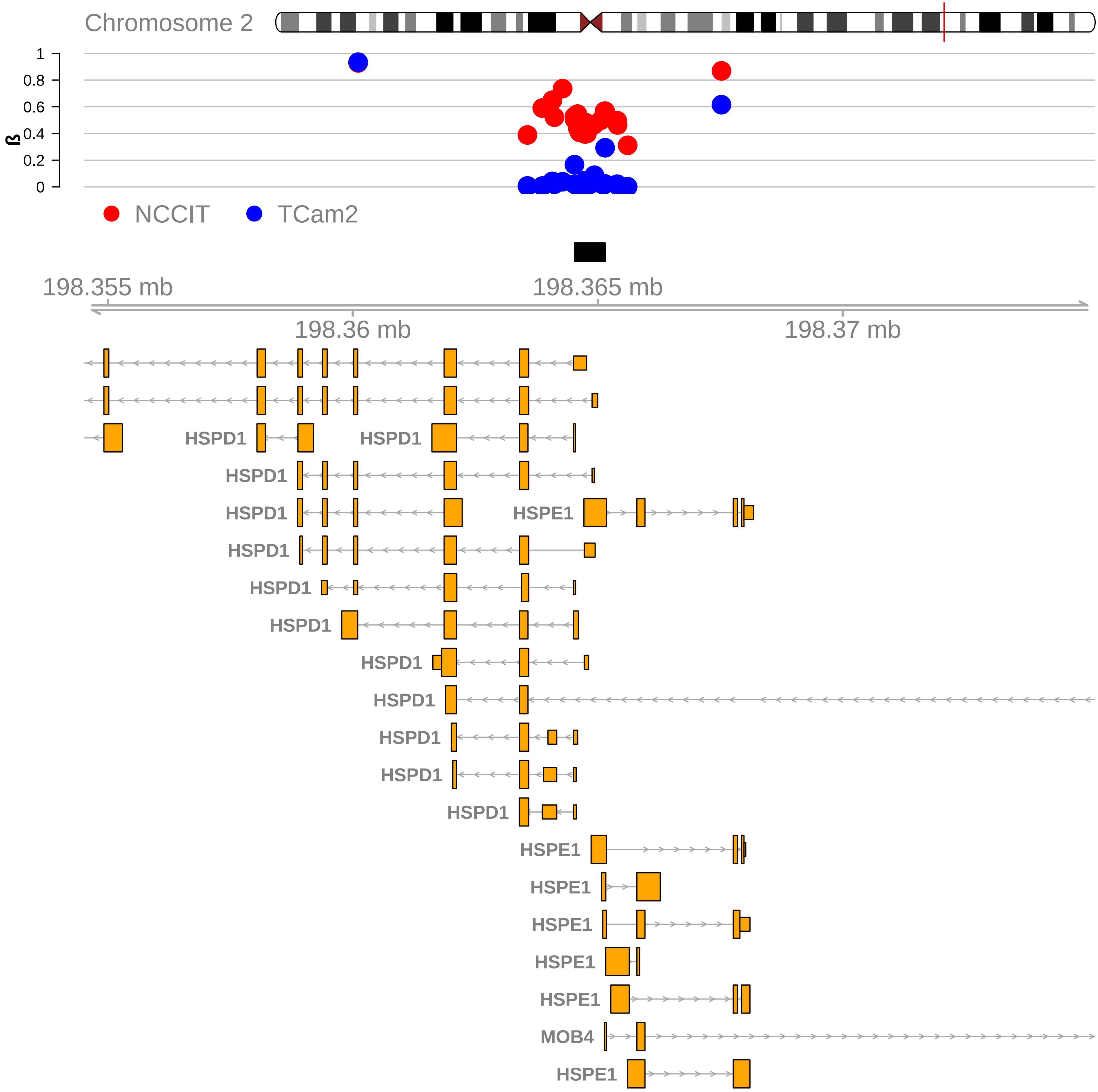

Chromosome 2
ß
1
0.8
0.6
0.4
0.2
0
NCCIT
TCam2
198.355 mb
198.365 mb
198.36 mb
198.37 mb
HSPD1
HSPD1
HSPD1
HSPD1
HSPE1
HSPD1
HSPD1
HSPD1
HSPD1
HSPD1
HSPD1
HSPD1
HSPD1
HSPE1
HSPE1
HSPE1
HSPE1
HSPE1
MOB4
HSPE1
